# Physical function and self-rated health status as predictors of mortality: results from longitudinal analysis in the ilSIRENTE study

**DOI:** 10.1186/1471-2318-8-34

**Published:** 2008-12-22

**Authors:** Matteo Cesari, Graziano Onder, Valentina Zamboni, Todd Manini, Ronald I Shorr, Andrea Russo, Roberto Bernabei, Marco Pahor, Francesco Landi

**Affiliations:** 1 Dipartimento di Scienze Gerontologiche, Geriatriche e Fisiatriche, Università Cattolica del Sacro Cuore, Roma, Italy; 2 Department of Aging and Geriatric Research, University of Florida – Institute on Aging, Gainesville, FL, USA; 3 Veterans Affairs Geriatric Research, Education and Clinical Center, Gainesville, FL, USA

## Abstract

**Background:**

Physical function measures have been shown to predict negative health-related events in older persons, including mortality. These markers of functioning may interact with the self-rated health (SRH) in the prediction of events. Aim of the present study is to compare the predictive value for mortality of measures of physical function and SRH status, and test their possible interactions.

**Methods:**

Data are from 335 older persons aged ≥ 80 years (mean age 85.6 years) enrolled in the "Invecchiamento e Longevità nel Sirente" (*ilSIRENTE*) study. The predictive values for mortality of 4-meter walk test, Short Physical Performance Battery (SPPB), hand grip strength, Activities of Daily Living (ADL) scale, Instrumental ADL (IADL) scale, and a SRH scale were compared using proportional hazard models. Kaplan-Meier survival curves for mortality and Receiver Operating Characteristic (ROC) curve analyses were also computed to estimate the predictive value of the independent variables of interest for mortality (alone and in combination).

**Results:**

During the 24-month follow-up (mean 1.8 years), 71 (21.2%) events occurred in the study sample. All the tested variables were able to significantly predict mortality. No significant interaction was reported between physical function measures and SRH. The SPPB score was the strongest predictor of overall mortality after adjustment for potential confounders (per SD increase; HR 0.64; 95%CI 0.48–0.86). A similar predictive value was showed by the SRH (per SD increase; HR 0.76; 95%CI 0.59–0.97). The chair stand test was the SPPB subtask showing the highest prognostic value.

**Conclusion:**

All the tested measures are able to predict mortality with different extents, but strongest results were obtained from the SPPB and the SRH. The chair stand test may be as useful as the complete SPPB in estimating the mortality risk.

## Background

Over the past two decades, there has been a growing recognition of the functional status assessment as a key factor in the evaluation of older persons[1]. This importance is mainly due to the large evidence that physical function measures are not only associated with clinical and subclinical age-related modifications[2,3], but are also able to predict future health-related events, including disability[4,5], institutionalization [6-8], and mortality[6,9].

Among the different possible options to evaluate the physical functioning of older persons, the use of specifically designed questionnaires aimed at evaluating how the subject interacts with the surrounding environment and identifying his impairments is one of the more commonly chosen. Best examples of this kind of tests are the Activities of Daily Living (ADL)[10], and Instrumental Activities of Daily Living (IADL)[11] scales, which were designed about 30–40 years ago. More recently, objective measures of physical performance and muscle strength have been developed to obtain objective estimates of the older persons' physical function. It has been shown that physical performance and muscle strength measures are able to identify more limitations in physical functioning than self-reported or subjective measures[12,13], and may be more useful for longitudinal evaluations because more sensible to changes[14]. Among these objective measures of physical function, the Short Physical Performance Battery (SPPB)[4], the 4-meter walking test[5], and the hand grip strength[15] are the most commonly used in clinical as well as research settings. Unfortunately, despite of the demonstrated critical role of physical function in the evaluation of older persons, the screening visit of an older person still mostly relies on self-reported questions (mainly due to time and/or space restrains commonly present especially in the clinical practice).

Similarly to physical function measures, self-rated health (SRH) has been shown to significantly predict negative outcomes (including disability[16] and mortality [17-20]). It has been explained that SRH might better capture the burden of clinical and subclinical conditions compared to the traditionally adopted measures of disease, or that positive self-ratings may mirror a general optimistic disposition[21] (consequently promoting a virtuous cycle with beneficial effects on neurological, immunological and endocrinological pathways[22]). The relationship between SRH and mortality has recently been shown to be independent of several potential confounders, including physical function (i.e., isometric muscle strength)[22].

However, some issues about the use of all these measures are still present and need clarification. Firstly, although all of the above-presented measures have shown to be predictive of mortality, a direct comparison among them for this outcome has not yet been formally conducted in literature. Secondly, physical performance and muscle strength measures (which on a broader extent represent markers of well-being) may interact with the self-perception of the health status in the prediction of events, but this hypothesis has never been explored. Investigating it may provide useful insights on the best way to manage all these screening instruments. It is noteworthy that evidence is particularly lacking for the very old persons. This aged group is the one in which evidence-based medicine is often very difficult to apply and clinical decisions are often driven by the subject's feelings. Therefore, all the screening instruments we plan to examine play a major rule in the determination of the frail older individual physical, functional, and biological reserves. Finally, the age-related decline in physical function due to the higher number of clinical and subclinical conditions may modify the predictive value of the commonly used measures of physical function as well as the self-perceived health status in the oldest old. Consequently, the clinical meaning of these markers may change in this age group.

In the present study, we hypothesized that 1) physical function and self-rated health (SRH) measures are predictive of negative health-related events in very old persons, and 2) a possible added effect of these instruments may allow a better prediction of events compared to when a single test is used. Therefore, we compared the predictive value for mortality of several measures of physical function (i.e. two measures of physical performance, the SPPB[4] and the 4-meter walking speed test; a marker of muscle strength, the hand grip strength; and two scales of disability, the ADL[10] and the IADL[11] scales), and a self-perceived measure of well-being (i.e. a SRH scale[23]) in a sample of very old persons (aged 80 years and older) enrolled in the "Invecchiamento e Longevità nel Sirente" (Aging and longevity in the Sirente geographic area, *ilSIRENTE*) study[24].

## Methods

We used baseline data from the ilSIRENTE, a prospective cohort study performed in the mountain community living in the Sirente geographic area (L'Aquila, Italy) and developed by the teaching nursing home Opera Santa Maria della Pace (Fontecchio, L'Aquila, Italy) in a partnership with local administrators and primary care physicians. Details of the design and methods of ilSIRENTE have been described elsewhere[24]. Briefly, potential study participants were identified by selecting from the Registry Offices every person born before 1^st ^January 1924 and still living in the municipalities involved in the study at the end of October 2003. A total of 364 participants were enrolled in the study. Participants' baseline assessments began in December 2003 and were completed in September 2004. Clinical interview and functional assessment were performed at the study clinics located in each town. Home visit was performed if participant was unable to reach the study clinic. Information was obtained by the participant or, if he/she was incapable, by a proxy. The Università Cattolica del Sacro Cuore (Roma, Italy) Ethical Committee approved the study protocol. All the participants signed an informed consent at the baseline visit.

The present analyses were conducted in 335 participants, after exclusion of 29 participants with missing data for the main variables of interest.

### The Minimum Data Set for Home Care (MDS-HC)

The Minimum Data Set for Home Care (MDS-HC) instrument[25] was administered to all study participants. The MDS-HC contains a variety of different, multi-item summary scales, exploring socio-demographics, clinical diagnoses, and physical function status. Besides, the MDS-HC includes information about an extensive array of signs, symptoms, syndromes, and treatments. The MDS items have shown an excellent inter-rater and test-retest reliability when completed by nurses performing usual assessment duties (average weighted Kappa = 0.8[26,27]). A questionnaire exploring family history, lifestyle, nutrition, physical activity, and other behavioral factors shared with the "Invecchiare in Chianti" (Aging in the Chianti geographic area, InCHIANTI) study[28] was also additionally administered.

### Mortality

Vital status of all the study participants was ascertained from the general practitioners, and confirmed by the National Death Registry until 24 months after the baseline visit. The follow-up time considered for the present analyses was calculated as the time from the date of baseline visit to the date of death (for participants who died during the follow-up), and censored to 24 months for participants who did not die during the study follow-up.

### Physical performance, muscle strength and functional status measures

#### Physical performance measures

Physical performance was assessed by the 4-meter walking speed and the Short Physical Performance Battery score. This latter measure is composed of three timed tests: 4-meter walking speed, balance, and chair stand tests[5]. Timed results from each test were categorized into 5-level variables ranging from 0 (worst performers) to 4 (best performers) according to well-established cut-points[5]. The sum of the results from the three categorized tests (ranging from 0 to 12) was used for the present analyses.

Walking speed was evaluated measuring the participant's usual gait speed (in m/sec) over a 4-meter course. The following cut-points were used to categorize the variable: <0.46 m/s, a score of 1; 0.46 to 0.64 m/s, a score of 2; 0.65 to 0.82 m/s, a score of 3; ≥ 0.83 m/s, a score of 4. Participants unable to complete the task were scored 0.

To assess the chair stand test, participants were asked to stand up from a chair with their arms folded across the chest five times in a row as quickly as possible. The time needed to complete the task was recorded. The following cut-points were used to categorize the variable: ≥ 16.7 seconds, a score of 1; 13.7 to 16.6 seconds, a score of 2; 13.6 to 11.2 seconds, a score of 3; and ≤ 11.1 seconds, a score of 4. Subjects unable to complete the test received a score of 0.

To assess the balance test, participants were asked to perform three increasingly challenging standing positions: side-by-side position, semi-tandem position, and tandem position. Participants were asked to hold each position for 10 seconds. Participants were scored as 1 if they were able to hold a side-by-side standing position for 10 seconds, but were unable to hold a semi-tandem position for 10 seconds; a score of 2 if they were able to hold a semi-tandem position for 10 seconds, but were unable to hold a tandem position for more than 2 seconds; a score of 3 if they were able to stand in tandem position for 3 to 9 seconds; and a score of 4 if they were able to hold the tandem position for 10 seconds. Participants unable to complete the test were scored 0.

#### Muscle strength measure

Muscle strength was assessed by hand grip strength measured by a dynamometer (North Coast Hydraulic Hand Dynamometer, North Coast Medical Inc, Morgan Hill, CA, USA). One trial for each hand was performed, and the result from the strongest hand was used in the present analyses. Hand grip strength has shown to be predictive of major health-related events in older persons[15,29].

#### Functional status measures

In the ilSIRENTE study, Basic and Instrumental Activities of Daily Living (ADL and IADL, respectively) scales were assessed as part of the MDS-HC instrument[26]. The assessor evaluated the participants' capacity to perform each task included in the ADL and IADL scales. Being the MDS-HC a comprehensive geriatric assessment tool aimed at 1) identifying the critical issues of the health status and care of older persons and 2) designing a specifically-tailored intervention plan, the impairment in each task was defined as the disability and/or the need of assistance in adequately performing the task. Therefore, all the ADL and IADL items were coded as "0" if the participant was independent in performing the specific task, or as "1" if supervision was required and/or the participant was completely dependent. The ADL scale (range 0–7, a higher number indicates higher impairment) is composed by the following tasks: eating, dressing, personal hygiene, mobility in bed, dressing, transferring (from/to bed, chair or stand position), use of the toilet. The IADL scale (range 0–7, a higher number indicates higher impairment) included: meal preparation, shopping, telephone use, housekeeping, responsability for medication intake, handling finances, use of transportation.

### Self-assessed health status

At the baseline visit, a single-item measure of SRH was administered to all the participants[23]. Subjects were asked to answer to the question ''How is your health in general?'' rating their status as ''Very Poor'', ''Poor'', ''Sometimes Good, Sometimes Poor'', ''Good'', or ''Very Good''. The relative score ranging from 1 (worst) to 5 (best) was used for the present analyses. SRH has shown to be a powerful predictor of mortality[17].

### Covariates

Covariates considered in the present analyses include: sociodemographic characteristics (age, gender, and smoking habit), body mass index (BMI), comorbidity, medications, and biological marker levels (albumin and total cholesterol). Body weight was measured with light clothes using a calibrated scale. Body height was measured using a standard stadiometer. BMI was defined as weight (in kilograms) divided by the square of height (in meters). The cognitive performance scale (CPS) was used to assess cognitive status[30]. The CPS has shown an excellent inter-rater and test-retest reliability when completed by nurses performing usual assessment duties[26]. The CPS score ranges from 0 (intact cognition) to 6 (severely impaired cognition). The following clinical diagnoses were assessed by a study physician on the basis of self- (or proxy-) reported history and clinical records review and considered in the adjusted analyses: coronary heart disease, congestive heart failure, cerebrovascular disease, diabetes, cancer, depression, dementia. A cumulative index of comorbidity defined by the number of clinical conditions was used for the present analyses. Standard determinations of total cholesterol and serum albumin concentrations were determined by using commercialy available kits suitable for use on Olympus 2700 instrumentation (Olympus, Milano, Italy). C-Reactive protein (CRP) concentrations were determined by a high sensitivity Enzyme-Linked ImmunoSorbent Assay kit (Bender MedSystems, Vienna, Austria). The CRP assay had a sensitivity of 3 pg/mL. The intra-assay coefficient of variation was 6.9%.

### Statistical analysis

Means (and standard deviations, SD), proportions (in percentage) were calculated to describe the main characteristics of the study sample. Unadjusted and adjusted proportional hazard models were performed to estimate the hazard ratios (HR, and 95% confidence intervals, 95%CI) of mortality (dependent variable) for physical performance, muscle strength, physical function, and SRH variables (independent variables). To permit direct comparisons of predictors, all the results are shown per SD increase of the measures. Kaplan-Meier survival curves for mortality were also performed according to physical performance and SRH groups. Receiver Operating Characteristic (ROC) curve analyses were also computed to estimate the predictive value of the independent variables of interest for mortality (alone and in combination) through the evaluation of the Areas Under the Curves (AUCs). A p value < 0.05 was chosen for statistical significance for all the present analyses. All the analyses were performed using SPSS software (version 13.0, SPSS Inc., Chicago, IL).

## Results

Main characteristics of the study sample population (n = 335; mean age 85.6 [SD 4.8] years) are presented in Table 1 according to vital status at the end of follow-up (mean length 1.8 [SD 0.5] years; 71 [21.2%] events). Compared to participants alive at the end of the follow-up, those who died were older and had a higher prevalence of congestive heart failure, cerebrovscular disease, depression, diabetes, and dementia. They also had lower BMI, albumin, and total cholesterol, and higher CRP concentration. For what concern the variables of interest for the present study, all the measures of physical function as well as the SRH score were significantly higher in participants alive at the end of the follow-up compared to cases.

**Table 1:** Main characteristics (mean ± standard deviation, or percentage) of the study sample according to mortality.

	**Death** (n = 71)	**No death** (n = 264)	**p**
*Sociodemographic*			
Age (years)	87.5 ± 5.2	85.0 ± 4.6	<0.001
Gender (women)	64.8	67.0	0.72
Education (years)	4.9 ± 1.1	5.2 ± 1.8	0.14
Current smoking	2.8	2.3	0.79
Body Mass Index (kg/m^2^)	24.4 ± 4.2	26.1 ± 4.5	0.003
Cognitive Performance Scale	1.6 ± 1.9	0.6 ± 1.3	<0.001
			
*Clinical conditions*			
Coronary artery disease	15.5	11.0	0.30
Congestive heart failure	15.5	3.4	<0.001
Cerebrovascular disease	14.1	1.9	<0.001
Cancer	8.5	3.4	0.07
Depression	35.2	23.9	0.05
Diabetes	36.6	25.0	0.05
Dementia	14.1	5.3	0.01
Number of clinical conditions	1.4 ± 1.2	0.7 ± 0.8	<0.001
			
*Biological markers*			
Albumin (g/dL)	4.1 ± 0.4	4.2 ± 0.3	0.001
Total cholesterol (mg/dL)	178.7 ± 38.0	203.6 ± 45.2	<0.001
C-reactive protein (mg/L)	5.1 (2.5–7.4)	2.5 (1.3–4.6)	<0.001
			
*Physical performance measures*			
4-meter walking speed	0.35 ± 0.30	0.55 ± 0.28	<0.001
SPPB score (0–12)	4.0 ± 3.3	7.1 ± 3.3	<0.001
Walk test (0–4)	1.3 ± 1.1	2.1 ± 1.2	<0.001
Chair stand test (0–4)	0.7 ± 1.1	1.8 ± 1.4	<0.001
Balance test (0–4)	2.0 ± 1.6	3.1 ± 1.3	<0.001
Hand grip strength (kg)	27.7 ± 14.4	33.2 ± 13.7	<0.001
*Functional status measures*			
ADL score	4.3 ± 2.2	6.2 ± 1.8	<0.001
IADL score	2.4 ± 2.5	4.7 ± 2.3	<0.001
*Self-rated health status*			
Self-Rated Health score	2.97 ± 0.89	3.48 ± 0.83	<0.001

SPPB: Short Physical Performance Battery; ADL: Basic Activities of Daily Living (range 0 [totally dependent] – 7 [totally independent]); IADL: Instrumental Activities of Daily Living (range 0 [totally dependent] – 7 [totally independent]).

Cognitive Performance Scale range: 0 (best) – 6 (worst); Self-Rated Health score range: 1 (worst) – 5 (best).

Results from unadjusted and adjusted proportional hazard models predicting mortality for all the variables of interest (per their SD increases) are shown in Table 2. Inverse and significant relationships of all the measures of physical function and SRH with mortality were found, even when models were adjusted for age and gender (all p values < 0.001). However, when additional potential confounders (i.e. number of clinical conditions and biological markers) were included into the models, the hand grip strength and the ADL score lost their statistical significance. In the final adjusted model, when also CRP concentration (log value) was included as covariate, the only SPPB and the SRH scores maintained their statistical significance in their association with mortality (HR 0.64, 95%CI 0.48–0.86; p = 0.003, and HR 0.76, 95%CI 0.59–0.97; p = 0.03, respectively).

**Table 2:** Proportional hazard models between measures of health status (per standard deviation increase) and mortality.

	**Unadjusted**	**Model 1**	**Model 2**	**Model 3**
	*HR (95%CI)*	*HR (95%CI)*	*HR (95%CI)*	*HR (95%CI)*
*Objective measures*				
4-meter walking speed	0.49 (0.38–0.63)^†^	0.53 (0.40–0.69)^†^	0.73 (0.54–0.99)*	0.77 (0.56–1.05)
SPPB score	0.44 (0.34–0.57)^†^	0.46 (0.36–0.60)^†^	0.62 (0.47–0.83)^†^	0.64 (0.48–0.86)^†^
SPPB Walk test	0.48 (0.36–0.64)^†^	0.52 (0.39–0.70)^†^	0.71 (0.52–0.97)*	0.73 (0.54–1.01)
SPPB Balance test	0.55 (0.44–0.68)^†^	0.58 (0.47–0.74)^†^	0.77 (0.60–1.00)	0.78 (0.60–1.01)
SPPB Chair stand test	0.38 (0.27–0.53)^†^	0.40 (0.29–0.56)^†^	0.51 (0.36–0.72)^†^	0.54 (0.38–0.76)^†^
Hand grip strength (kg)	0.55 (0.43–0.71)^†^	0.57 (0.44–0.75)^†^	0.77 (0.58–1.04)	0.78 (0.58–1.05)
ADL score	0.54 (0.45–0.65)^†^	0.58 (0.48–0.71)^†^	0.77 (0.59–1.00)	0.81 (0.62–1.06)
IADL score	0.48 (0.38–0.61)^†^	0.51 (0.40–0.67)^†^	0.70 (0.50–0.99)*	0.77 (0.54–1.09)
SRH score	0.62 (0.50–0.77)^†^	0.65 (0.52–0.80)^†^	0.78 (0.60–0.99)*	0.76 (0.59–0.97)*

* p < 0.05; † p < 0.01

SPPB: Short Physical Performance Battery; ADL: Basic Activities of Daily Living; IADL: Instrumental Activities of Daily Living; SAHS: Self-assessed health status.

*Standard deviations: *4-meter walking speed 0.29714; SPPB score 3.50676; SPPB Walk test score 1.22735; SPPB Balance test score1.45514; SPPB Chair stand test score 1.42872; Hand grip strength 14.28956 kg; Basic Activities of Daily Living 2.19641; Instrumental Activities of Daily Living 2.51375; Self-rated health 0.86923

*Model 1: *Adjusted for age and gender.

*Model 2: *Adjusted for Model 1 + body mass index, cognitive performance scale, number of clinical conditions, albumin, total cholesterol.

*Model 3: *Adjusted for Model 2 + C-reactive protein (log value)

Separate partially adjusted models (for statistical power reasons; Model 1 adjustment) were also performed using the categorical variables (ranging from 0 to 4) for each subtask of the SPPB as independent variable of interest. Statistically significant and positive associations were reported between all the SPPB tasks and survival (all p for trend < 0.001). At the 4-meter walking speed test, participants scoring 1, 2, 3, and 4 had 56.3%, 63.6%, 83.1%, and 85.7% lower mortality risk compared to the reference group (i.e. subjects scoring 0), respectively. Similar findings were also found for the chair stand (45.9%, 72.8%, 92.5%, and 86.6%, respectively) and the balance (47.0%, 58.0%, 69.7%, and 79.2%, respectively) tests.

Analyses were also conducted to evaluate which of the three subtasks composing the SPPB score was the most strongly associated with mortality. Results showed that the chair stand test was the only significantly associated with mortality, while only borderline significances were found for the balance, and the 4-meter walk test. No significant interaction for the prediction of mortality was found between physical function measures and SRH. No significant gender interaction was found between all the independent variables of interest and mortality (all p values for interaction terms >0.3).

Since the chair stand test was the SPPB subtask showing the strongest association with mortality, secondary analyses were performed to evaluate the possible existence of an additive predictive value for mortality of this SPPB component with the SRH.

No significant differences were found among AUCs designed by ROC curve analyses for mortality when the chair stand test (AUC 0.725, 95%CI 0.661–0.789), the SRH (AUC 0.656, 95%CI 0.582–0.730), and their combination (AUC 0.751, 95%CI 0.686–0.816) were tested. Similar findings were reported when testing the SPPB score (AUC 0.743, 95%CI 0.679–0.806), and its combination with the SRH (AUC 0.749, 95%CI 0.683–0.814).

**Figure 1:**
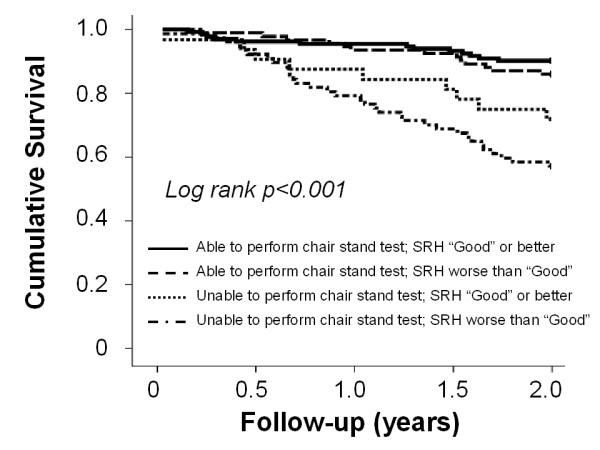
**Kaplan-Meier survival curves for mortality according to the ability to perform the chair stand test and self-rated health (SRH) score**.

The chair stand test was then categorized according to the ability (n = 226, 67.5%) or not (n = 109, 32.5%) to perform the task. The SRH score was categorized according to the median value in two groups (i.e. SRH score ≤ 3: n = 170 [47.9%]; SRH score >3: n = 165 [52.1%]). Figure 1 shows results from Kaplan-Meier survival curves for mortality according to physical performance and SRH groups. Participants able to complete the chair stand test were significantly less likely to die compared to those with poor physical performance (p < 0.001). No significant differences were found 1) among participants able to complete the chair stand test, or 2) among participants unable to complete the chair stand test, according to the SRH status groups (pairwaise comparisons p = 0.47, and p = 0.17, respectively). An adjusted multivariable proportional hazard model (Table 3) confirmed these findings, showing that participants unable to complete the chair stand test and with worse SRH had a higher risk of mortality (HR 2.36, 95%CI 1.16–4.79; p = 0.02) compared to the reference group (i.e. able to complete the chair stand test and SRH score >3), even after adjustment for all the potential confounders. Consistent results were obtained when the overall SPPB score was tested in combination with the SRH.

**Table 3:** Results from a single multivariable proportional hazard model* exploring the relationship of physical performance and self-rated health with mortality.

	***N/N (%)***	***Hazard Ratio (95% Confidence Interval)***
SPPB Chair stand test > 0, SRH > 3	14/133 (10.5)	1 (Reference group)
SPPB Chair stand test > 0, SRH ≤ 3	14/93 (15.1)	1.02 (0.46–2.25)
SPPB Chair stand test = 0, SRH >3	9/32 (28.1)	1.45 (0.57–3.74)
SPPB Chair stand test = 0, SRH ≤ 3	34/77 (44.2)	2.36 (1.16–4.79)

n/N: number of events/number of participants; SPPB: Short Physical Performance Battery; SRH: Self-rated health

*Adjusted for age, gender, body mass index, cognitive performance scale, number of clinical conditions, albumin, total cholesterol, C-reactive protein (log value)

## Discussion

In the present study, we compared the predictive value for mortality of two physical performance measures (i.e. 4-meter walk test, and the SPPB score), a measure of muscle strength (i.e. hand grip), two measures of disability (i.e. ADL and IADL scores) and a self-reported measure of well-being (i.e. a SRH scale). Our results from unadjusted and partially adjusted analyses showed that all the tested physical function variables were able to predict mortality. However, the SPPB score was the strongest predictor of overall mortality in these very old community-dwelling subjects, even after considering several socio-demographic, clinical, and biological confounders. A lower, but still significant, predictive value was only showed by the SRH measure. Among the three subtasks of the SPPB, the chair stand test was the one showing the highest prognostic value. The combination of the chair stand test and the SRH score did not provide significant additional benefits in predicting mortality. In fact, participants with a good physical performance had a lower risk of dying compared to those with poor performance, independently of their self-perceived health status. Moreover, no significant differences were reported when comparing the AUC designed by ROC curve analyses for mortality. However, when the chair stand test and the SRH results were combined, the selection of the participants poorly scoring at both tests, led to the identification of a smaller number of subjects characterized by the highest risk of mortality compared to participants with good physical performance and SRH.

Previous studies have already explored the relationship existing between objective and self-reported measures of physical function for major health-related events[31,32]. Moreover, the strong relationship between physical performance tests and negative health-related outcomes in the elderly has already been documented[4,6,33,34]. However, besides of being confirmatory of previous findings showing the importance of physical performance in older persons, our study still adds some novel contributions to the topic.

To our knowledge, a direct comparison of the predictive value for mortality of different screening instruments (particularly aimed at the evaluation of the physical function and the health status) is not yet available in literature, especially among very old subjects. This comparison led to the identification of the SPPB (in particular, of the chair stand test subtask) and the SRH as the best predictors of mortality. It is noteworthy that both these instruments are quick and inexpensive measures whose implementation in clinical settings may not be particularly honerous in terms of training, costs, and time.

Our results did not show evidence of a statistical interaction between SPPB, SRH and mortality. However, the survival analyses we performed seem to suggest such interaction. In fact, the SPPB score was able to discriminate participants with higher mortality risk regardless of their SRH status. This was not evident for the SRH instrument, which tended to discriminate individuals at higher risk only among those with poor physical performance. This finding may support the use of SPPB (and physical performance measures in general) as optimal screening tools for older persons, independently of their health status.

Our analyses of the three subtests composing the SPPB demonstrated that the chair stand test was more strongly associated with the mortality outcome than the walk and balance tests. Moreover, the chair stand test was able to predict mortality in a very similar way to the complete SPPB score. This may suggest that the adoption of this only subtask in those settings with time and/or space restrains might already be sufficient to identify older persons at risk of events. In the attempt to facilitate the possible implementation of this test in the clinical setting, our secondary analyses tested a dichotomous variable of the chair stand test defined as the ability or not to stand up from the chair five times in a row. The adoption of this single SPPB subtask as screening tool for older persons may be very easy to implement, even more than a walking speed test. Interestingly, Ensrud and collesgues recently proposed a frailty index including the inability to rise from a chair 5 times without using arms as a component criterion[35]. Authors compared the predictive value of this new index to that of the more commonly used (but more complex) Fried and colleagues' one[36], reporting similar results. Our results showing the higher prognostic value of the chair stand test in comparison with the other SPPB subtasks is not completely in line with the sparse previous evidence. In fact, the few studies available on the topic suggest that the walking speed is the most sensitive subtask of the SPPB in predicting incident disability[4,37], and mortality[38]. A possible explanation to the different results we found might be the older age of our sample population. It might be that the three subtests composing the SPPB may present different age-related declines. If the 4-meter walk test is more prematurely affected by aging (and the related underlying conditions), a "floor" effect may limit the predictive value of it in favor of a possible more stable test (i.e. chair stand test).

The predictive value for mortality of SRH, independently of health risk factors is well-demonstrated in literature[17]. Several explanations to this relationship have been provided. It is possible that SRH may better capture the burden of diseases and symptoms. Another explanation might be related to the wider spectrum of information (inclusive of personal sensations) that a person can describe when self-reporting the own health status, and which may partially be excluded by "external" evaluations[39].

Significant results in the prediction of mortality were reported by the hand grip strength, and the IADL and ADL scales only in the unadjusted and partially adjusted models. It is noteworthy that the predictive values of the hand grip strength, ADL, and IADL scores for mortality were strongly weakened by inclusion in the statistical models of clinical conditions and, later, CRP concentrations. Consistent results have previously been reported in studies testing the associations of these measures with comorbidity[32], health status[14], and incident health-related events[33,40]. The hand grip strength is a standardized measure of a specific muscular district strength which is generalized to the overall individual muscular functioning[15,41]. On the other hand, the SPPB requires a good overall physical functioning of the subject to be successfully completed. The ADL and IADL scales are designed to evaluate the ability of a subject to interact with the surrounding environment and independently accomplish crucial tasks of life[10,11]. Thomas and colleagues recently showed that objective measures of physical performance are able to improve the assessment of functional status provided by subjective measures of physical function in older persons[13]. Therefore, it is likely that the SPPB is able to capture a wider scope of information from different sources related to physical functioning than hand grip strength, and ADL and IADL scales. In this context, it may also not be surprising that a general SRH measure is more strongly associated to mortality than specifically-aimed subjective screening tests (i.e. ADL and IADL scales) or too sectorial measures (i.e. hand grip strength).

Our study presents some limitations. The limited sample size may have influenced some of our results, potentially leading to type I errors. However, the risk of false negative results may be limited due to the overall consistency of our findings (even with previous reports). Our sample population was composed by older community-dwelling persons aged 80 years and older. Further studies confirming our findings, and extending them to different age groups, settings and populations are needed. Third factors not considered in our study (e.g. body composition), and potentially explaining (at least partly) our results may represent a further limit of the present analyses.

## Conclusion

Our study shows that all the tested measured are able to predict mortality with different extents. However, the only which are not influenced by sociodemographic, clinical, and biological factors in their prediction are the SPPB and the SRH. The chair stand test may be as useful as the complete SPPB in estimating the mortality risk, and the testing of the only ability to perform it may already be sufficient to provide useful prognostic information.

## Competing interests

The authors declare that they have no competing interests.

## Authors' contributions

MC conceived and designed the study, carried out the data analysis and interpreted the results, drafted the manuscript, and gave the final approval. GO conceived and designed the study, carried out the data analysis and interpreted the results, helped in drafting the manuscript, provided critical review of the manuscript, and gave the final approval. VZ conceived and designed the study, was involved in the data acquisition, helped in the interpretation of the data, provided critical review of the manuscript, and gave the final approval. TM helped in the interpretation of the data, provided critical review of the manuscript, helped in drafting the manuscript, and gave the final approval. RIS helped in the interpretation of the data, provided critical review of the manuscript, and gave the final approval. AR conducted the acquisition of the data, provided critical review of the manuscript, and gave the final approval. RB helped in the interpretation of the data, provided critical review of the manuscript, and gave the final approval. MP was involved in the conception and design of the study, helped in the interpretation of the data, provided critical review of the manuscript, and gave the final approval. FL conceived and designed the study, was responsible for the acquisition of the data, helped in the analysis and interpretation of the data, provided critical review of the manuscript, and gave final approval.

## Pre-publication history

The pre-publication history for this paper can be accessed here:


